# The screening of immune-related biomarkers for prognosis of lung adenocarcinoma

**DOI:** 10.1080/21655979.2021.1911211

**Published:** 2021-04-17

**Authors:** Zhonghui Liu, Dan Sun, Qing Zhu, Xinmin Liu

**Affiliations:** a Department of Geriatrics, Peking University First Hospital, Beijing, China; b Institutes of Biomedical Sciences, Shanghai Medical College of Fudan University, Shanghai, China

**Keywords:** Lung adenocarcinoma (LUAD), bioinformatics analysis, immune infiltration

## Abstract

Lung adenocarcinoma (LUAD) accounts for a frequently seen non-small cell lung cancer (NSCLC) histological subtype, and it is associated with dismal prognostic outcome. However, the benefits of traditional treatment are still limited, and the efficacies of immunotherapy are quite different. Therefore, it is of great significance to identify novel immune-related therapeutic targets in lung adenocarcinoma. In this study, we identified a set of immune-related biomarkers for prognosis of lung adenocarcinoma, which could provide new ideas for immunotherapy of lung adenocarcinoma. Datasets related to LUAD were filtered from the GEO database. The appropriate packages were used to identify differentially expressed genes (DEGs) and to carry out enrichment analysis, followed by the construction of prognostic biomarkers. The Kaplan-Meier (K-M) curves were plotted to analyze patient survival based on hub genes. Associations between the expression of selected biomarkers and six types of tumor-infiltrating immune cells were evaluated based on the online tool TIMER. After analyzing five GEO datasets(GSE32867, GSE46539, GSE63459, GSE75037 and GSE116959), we discovered altogether 67 DEGs, among which, 15 showed up-regulation while 52 showed down-regulation. Enrichments of integrated DEGs were identified in the ontology categories. CAV1, CFD, FMO2 and CLEC3B were eventually selected as independent prognostic biomarkers, they were correlated with clinical outcomes of LUAD patients. Moreover, a positive correlation was observed between biomarker expression and all different types of immune infiltration, and the expression level of the four biomarkers was all positively related to macrophage.

## Graphical abstract

**Figure:**
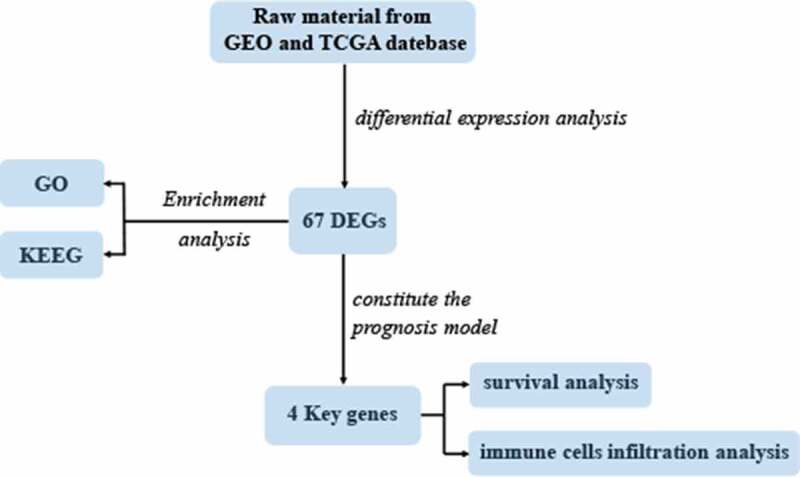

## Introduction

Lung cancer (LC) represents a major reason leading to cancer-associated mortality in the world because it has a poor survival [1, 2]. Non–small cell lung cancer (NSCLC) occupies approximately 85% LC cases, among which, lung adenocarcinoma (LUAD) represents the most commonly seen NSCLC histological subtype [3]. More crucially, approximately 70% LC cases are diagnosed at the advanced or metastatic stage [4]. However, conventional treatments for advanced lung cancer has limited effect. Although the combination therapies have been substantially developed, LUAD patients still have poor prognosis, with a < 20% 5-year survival [5]. There is an urgent need for better and lasting treatment for advanced lung cancer.The emergence of immunotherapy and its excellent effect provide a promising direction for the treatment of lung cancer [6]. For example, immune checkpoint inhibitors (ICIs) as well as adoptive cell transfer (ACT) can attain sustained clinical response. However, there are many problems to be considered in the current immunotherapy, for example, these treatments have distinct therapeutic effects and are only beneficial for certain cancer cases [7]. In addition, potential side effects, such as autoimmune response or cytokine release syndrome, also prevents further clinical application of current immunotherapy for lung cancer.

Therefore, it is of great significance to elucidate the molecular mechanisms of the occurrence and progression of LUAD, especially for the elucidation of the immunophenotypes of tumor-immune interactions and identification of novel immune-related therapeutic targets in LUAD. A comprehensive analysis of immune infiltration in tumors is helpful to clarify the mechanism of tumor immune escape, thus providing an opportunity for the development of new therapeutic strategies. Microarray technology has become a powerful tool for studying the differential expression of genes related to the carcinogenesis and progression of LUAD, which made it possible to analyze the tumor microenvironment and provide an opportunity to analyze the functional diversity of tumor infiltrating immune cells. In this study, we use a variety of bioinformatics methods to find immune-related biomarkers for prognosis of lung adenocarcinoma.

## Methods

### Data collection

Datasets related to LUAD were filtered against the GEO database (http://www.ncbi.nlm.nih.gov/geo) using the following Mesh terms, including ‘lung neoplasms’ and ‘human’, study type ‘Expression profiling by array’, organism ‘Homo sapiens’, together with samples count ‘Higher than ten’. Five microarray datasets were selected, including GSE32867, GSE46539, GSE63459, GSE75037, GSE116959. Raw material collection and processing were completed through background correction based on the robust multi–array average expression measure (RMA), normalization as well as log2 transformation. Table 1 presents the details of data. Additionally, we used the ‘TCGAbiolinks’ R package to obtain the sample expression profiles and related clinical data from The Cancer Genome Atlas (TCGA) database (https://gdc.cancer.gov).

**Table 1.** Characteristics of GEO datasets included in the study

Dataset ID	Platform ID	Country/Region	Number of Samples	
			Tumor	Normal
GSE32867	GPL6884	USA	86	85
GSE46539	GPL6883	Taiwan	115	115
GSE63459	GPL6883	USA	33	32
GSE75037	GPL6884	USA	83	83
GSE116959	GPL17077	France	57	11

GSE, Gene Expression Omnibus Series; GPL, Gene Expression Omnibus Platform

### Identification of DEGs

The ‘limma’ and ‘DESeq2ʹ R package were used to perform differential expression analysis with the standard comparison mode between two experimental conditions in each GEO data set [8]. P values and log2 fold change (FC) of duplicate genes were averaged. All gene lists sorted by logFC were integrated through the ‘RobustRankAggreg (RRA)’ R package [9]. Significant DEGs were defined as those with adjusted P < .01 and |log_2_ FC| > 2 in the final aggregated gene set.

### Enrichment analysis

Firstly, we used ‘clusterprofiler’ function in package for Gene Ontology (GO) as well as Kyoto Encyclopedia of Genes and Genomes (KEGG) analysis [10]. The 10 most significant biological process (BP), cellular component (CC) along with molecular function (MF) terms for DEGs together with the 3 most significant signal transduction KEGG pathways are displayed in the bubble charts. Then, we conducted association analysis to identify DEG enrichments in the following ontology categories: DisGeNET, PaGenBase and TRRUST using Metascape (http://www.metascape.org) [11-13].

### Construction of prognostic biomarkers

In the TCGA-LUAD dataset, the Bioconductor package ‘org.Hs.eg.db’ was used to convert Entrez IDs into gene symbols to match the names of DEGs we have found. Biomarkers for predicting LUAD prognosis were selected from candidate DEGs based on LASSO Cox regression analysis. By the use of R package ‘glmnet’, we applied the model in potential gene expression matrix and screened the best penalty parameter lambda for calculating the coefficients of all genes used to constitute the prognosis model. Besides, we built the multivariate Cox regression model based on the expression profiles of screened biomarkers and the clinicopathological variables (including age, gender, TNM stage, tumor stage, smoking status). Besides, the forward stepwise strategy was applied in selecting the most significant factors to independently predict prognosis. A forest plot was drawn to show P values, HRs and 95% CIs of eventually significant variables through the ‘forestplot’ package. Two scatterplots were plotted to describe the distribution of risk score and survival time based on the optimal cutoff to divide risk group. A heatmap was drawn to check the relationships between the genes and risk stratification. In accordance with the above results, we established a nomogram to predict the 1-, 3- and 5-year OS by using the ‘regplot’ and ‘rms’ R package. Besides, we used calibration curves to measure nomogram performance in which the closeness was investigated between the OS predicted by our nomogram and the real values. As a reference, diagonal represented the best prediction.

### Validation and survival analysis of biomarkers

The online database Gene Expression Profiling Interactive Analysis (GEPIA) (http://gepia.cancer-pku.cn/index.html), which covers extensive samples from 33 different cancer types, was adopted for investigating the differentially expressed biomarkers between tumor and non-tumor tissues [14]. Independent samples one-way analysis of variance (ANOVA) was used to assess whether gene expression distinguished among tumor stages. The tumor samples derived from TCGA-LUAD cohort and normal tissues came from TCGA-LUAD dataset and GTEx. In terms of survival analysis, those TCGA-LUAD samples were classified as 2 groups according to the optimal separation threshold of every hub gene, so as to draw the K-M survival curves by the use of the UALCAN online source (https://ualcan.path.uab.edu/index.html) [15].

### Correlations between biomarker expression and tumor-infiltrating immune cells

Associations between the expression of selected biomarkers and six tumor infiltrating immunocyte types were assessed using the TIMER online approach (https://cistrome.shinyapps.io/timer) [16]. Furthermore, the latest version TIMER2 (http://timer.cistrome.org) was used to obtain correlation matrices in which tumor infiltration was calculated through many different methods, such as EPIC, CIBERSORT and XCELL [17]. Heatmaps were drawn to visualize the relationships using the ‘pheatmap’ R package.

### Statistical analysis

The online web resources and R 3.6.1 were utilized for statistical analysis. The mRNA expression levels were compared between tumor and non-tumor samples by student’s t-test, whereas the differential expression was compared by one-way ANOVA across different tumor stages. Differences in survival were analyzed by the KM curves through log-rank test. Each assay was repeated thrice. A difference of P < .05 indicated statistical significance (*, P < .05).

## Results

In this study, we identified a set of immune-related biomarkers for prognosis of lung adenocarcinoma, which could provide new ideas for immunotherapy of lung adenocarcinoma. After analyzing five GEO datasets(GSE32867, GSE46539, GSE63459, GSE75037 and GSE116959), we discovered altogether 67 DEGs, among which, 15 showed up-regulation while 52 showed down-regulation. CAV1, CFD, FMO2 and CLEC3B were eventually selected as independent prognostic biomarkers, they were correlated with clinical outcomes of LUAD patients. Moreover, a positive correlation was observed between biomarker expression and all different types of immune infiltration.

### 67 DEGs were identified from five datasets

**Figure 1.**
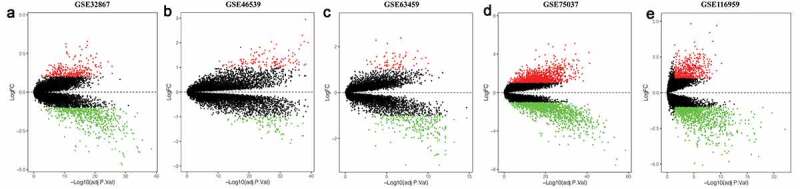
Differentially expressed genes between the two groups of samples in each dataset. (a) GSE32867, (b) GSE46539, (c) GSE63459, (d) GSE75037, (e) GSE116959. Red and green dots stand for up-regulated and down-regulated genes, respectively, while black dots stand for insignificant genes

**Figure 2.**
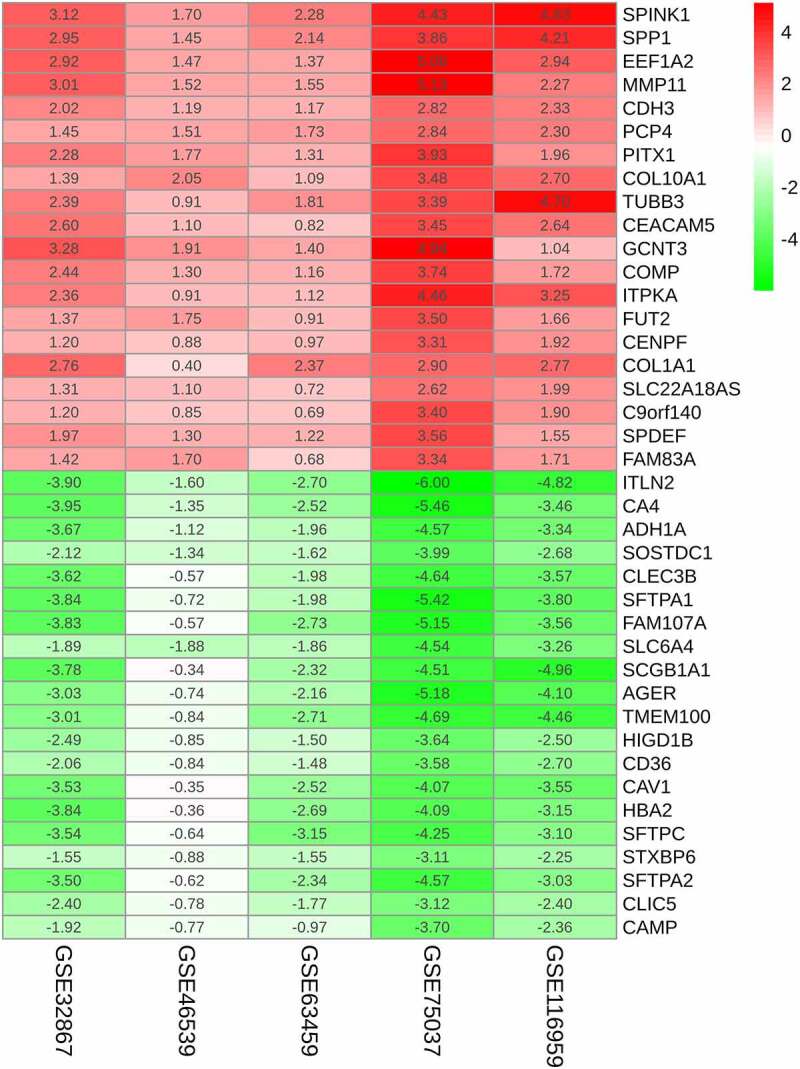
The RRA-based identification of potent DEGs. Heatmap showing the 20 most significantly up-regulated and down-regulated DEGs identified from the GEO series. The rows and columns stand for DEGs and datasets, respectively. The change in color from red to green indicated the change from up-regulation to down-regulation. Numbers within the box are the logarithmic FCs

Totally, five chip expression datasets related to LUAD were selected to identify DEGs, including GSE32867, GSE46539, GSE63459, GSE75037 and GSE116959. The DEGs identified in each dataset are shown in Figure 1. We sorted these 5 expression matrices according to the logarithmic fold change (log_2_ FC) of endogenous references, and incorporated them to select the significant DEGs. The 20 DEGs with the most significant up-regulation and down-regulation are displayed in the heatmap (Figure 2). Clearly, matrix metalloproteinase 11(MMP11) showed the most significant up-regulation level (log_2_ FC = 5.13), whereas intelectin-2(ITLN2) showed the most significant down-regulation level (log_2_ FC = −6.00). Under the criteria that adjusted P value is less than 0.01 and |log_2_ FC| more than 2, 67 integrated DEGs were eventually identified based on the RRA analysis, including 15 upregulated and 52 downregulated DEGs.

### Functional enrichment analysis of DEGs

**Figure 3.**
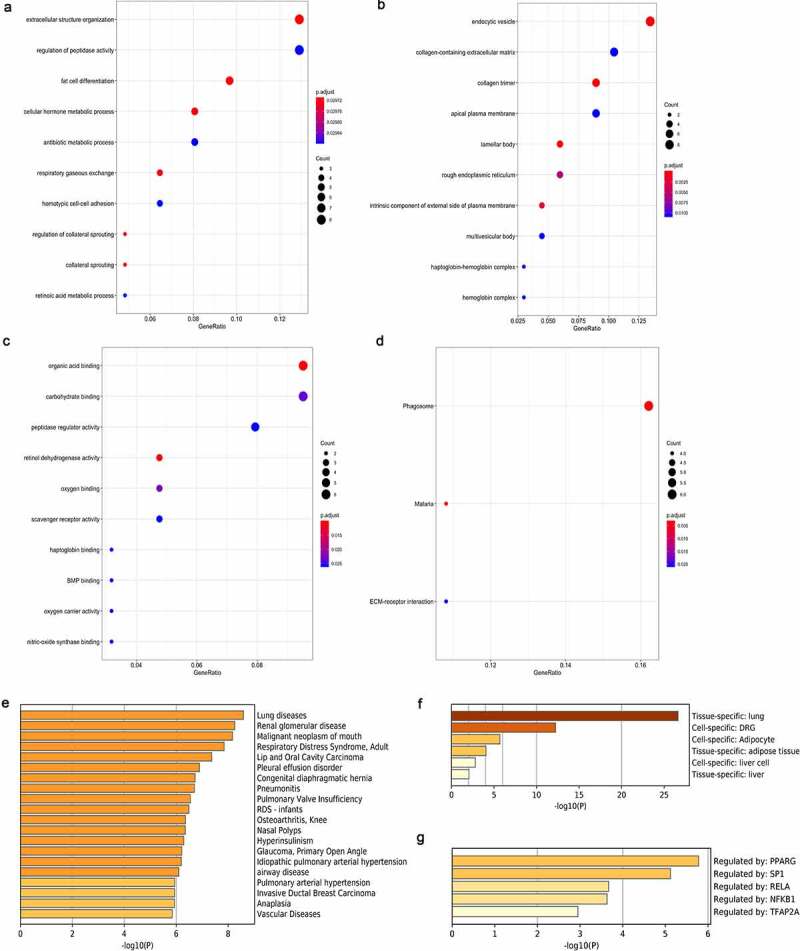
Enrichments of integrated DEGs are identified in the ontology categories. (a) Ten most significant BP terms. (b) Ten most significant CC terms. (c) Ten most significant MF terms. (d) Three most significantly enriched KEGG pathways. (e) The top 20 enriched DisGeNET terms. (f) The top 6 enriched PaGenBase terms. (g) The top 5 enriched TRRUST terms

GO analysis was conducted to examine the significantly integrated DEGs. Among them, the 3 most significant BP terms included extracellular structure organization, regulation of peptidase activity and fat cell differentiation. As for CC terms, endocytic vesicle, collagen-containing extracellular matrix and collagen trimer were the top three most significant ones. Besides, with regard to MFs, organic acid binding, peptidase regulator binding and carbohydrate binding were the most significant ones. As for KEGG pathways, those 6 DEGs were mostly associated with phagosome. In addition, there were four genes enriched in malaria and ECM-receptor interaction, respectively (Figure 3(a–d)). Interestingly, DEGs were also most significantly enriched in lung diseases regarding the ontology category DisGeNET, a gene-disease association database that contains the greatest free collection of variants and genes. The Pattern Gene Database (PaGenBase) is also an openly accessible database, which provides data for pattern genes (selective genes, specific genes, repressed genes and housekeeping genes) for altogether 11 model organisms detected based on gene expression profiling data under different physiological conditions. In this database, lung cancer is also the most commonly seen tissue-specific malignancy. The result of enrichment analysis based on the TRRUST database revealed that peroxisome proliferator-activated receptor gamma (PPARG) was the most significant transcriptional regulatory approach (Figure 3(e–g)).

### Four genes were selected as biomarkers with prognostic value

Biomarkers with prognostic value were selected from integrated DEGs by adopting the LASSO Cox model. Figure S1 presents the alterations of LASSO partial likelihood deviance along with coefficients with ln lambda. As a result, there were 22 genes when lambda reached the optimal value.

**Figure 4.**
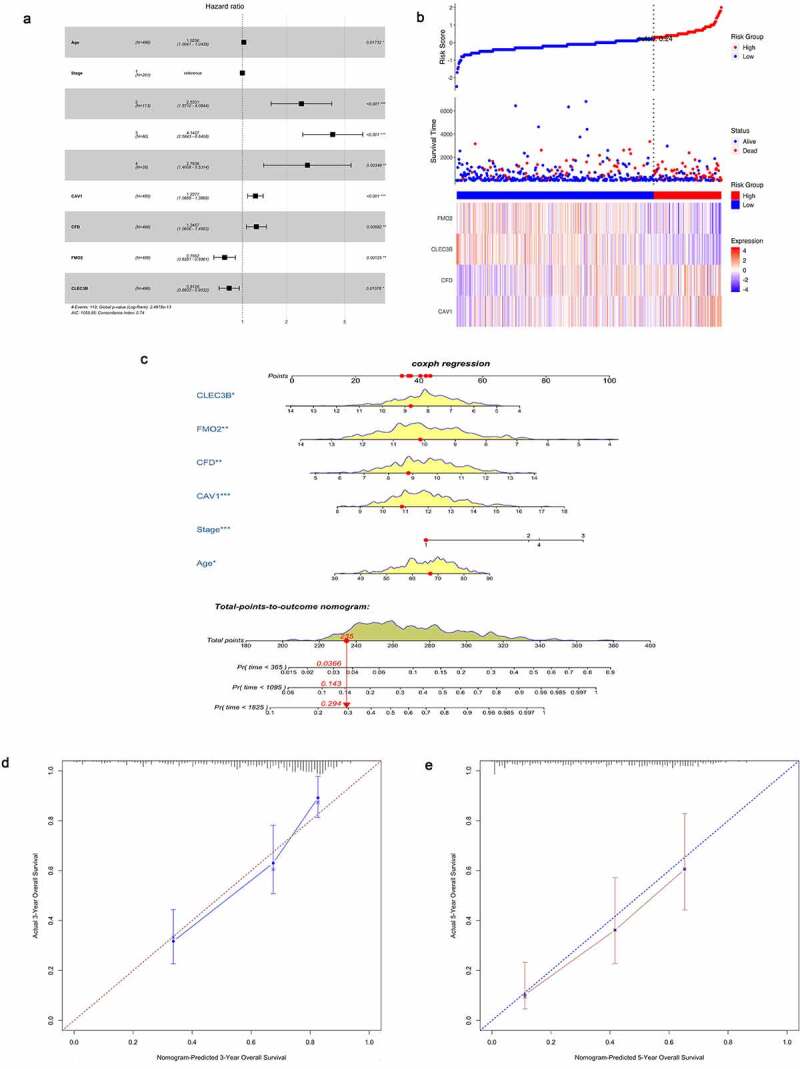
Construction of biomarkers with prognostic value. (a) Forest plot showing the eventual model established using the forward stepwise strategy. (b) Associations of the expression of biomarkers with survival for LUAD cases. (c) Nomogram for predicting 1-, 3-, and 5-year survival. (d) Calibration curve of the nomogram that predicted 3-year OS. (e) Calibration curve of the nomogram that predicted 5-year OS

These genes were considered as candidate biomarkers and included in the multivariate Cox regression model in which clinicopathological characteristics were covariates. The top one gene was selected from forward stepwise selection to the model. The result revealed that Caveolin1 (CAV1), complement factor D (CFD), flavin-containing monooxygenase 2 (FMO2) and C-type lectin domain family 3 member B (CLEC3B) were eventually selected as independent prognostic biomarkers. Age and tumor stage were also considered significantly in the model. The forest plot disclosed that the expression of CAV1 and CFD was positively correlated with clinical outcomes of LUAD patients while the expression of FMO2 and CLEC3B showed negative association (Figure 4(a)). The similar relationships between the expression and risk stratification were observed from (Figure 4(b)). We further combined the expression of four prognostic biomarkers with age and tumor stage to build a nomogram that visualized their prognostic value in predicting overall survival at 1, 3 and 5 years (Figure 4(c)). Moreover, calibration curves indicated that the nomogram had good prediction accuracy for 3- and 5-year overall survival (Figure 4(d,e)).

### Validation of prognostic biomarkers

**Figure 5.**
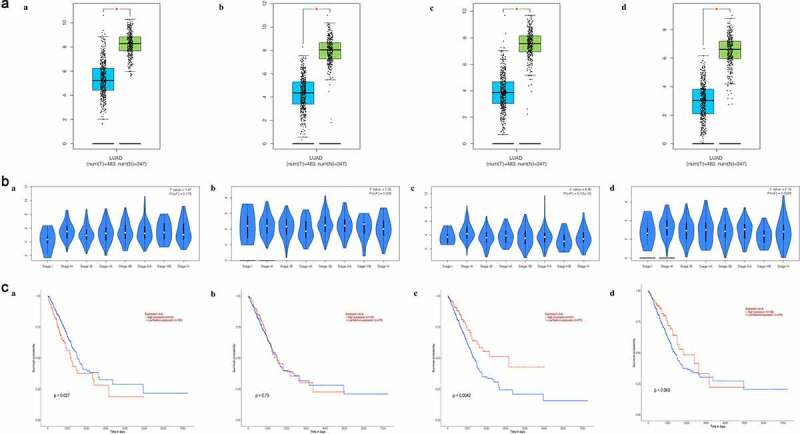
Biomarker validation. (a) Differential expression between tumor and matched non-carcinoma samples. (b) Expression in LUAD samples with different tumor stages. (c) Association between expression and overall survival time. (a) CAV1. (b) CFD. (c) CLEC3B. (d) FMO2

Noteworthily, the expression of each biomarker markedly decreased within tumor tissues in comparison with the matched non-carcinoma tissues (P < 0.001). Among a variety of tumor stage, the expression of FMO2 and CLEC3B differed significantly. The result of survival analysis suggested that CAV1 up-regulation and CLEC3B down-regulation indicated the dismal prognosis. (Figure 5).

### Association of biomarker expression with immune infiltration

**Figure 6.**
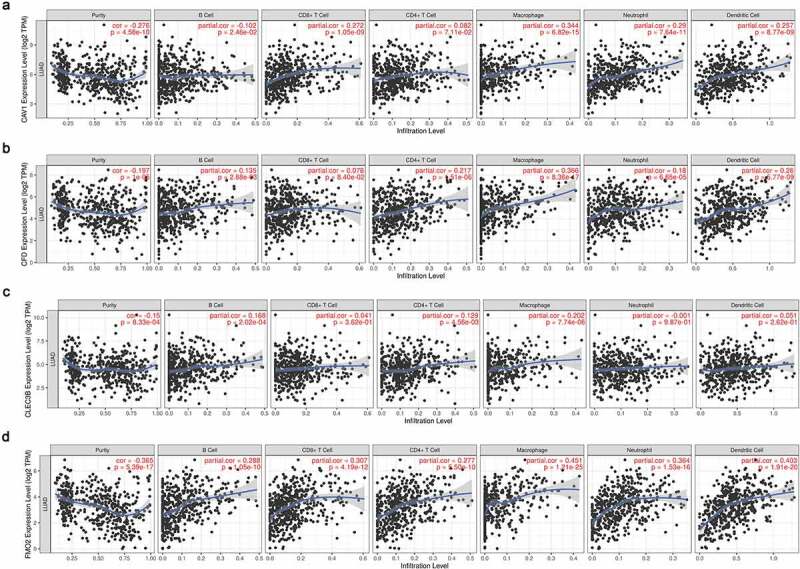
Relationship between the expression of biomarkers and immune infiltration degrees in LUAD. (a) CAV1. (b) CFD. (c) CLEC3B. (d) FMO2. P < 0.05 denotes significance

**Figure 7.**
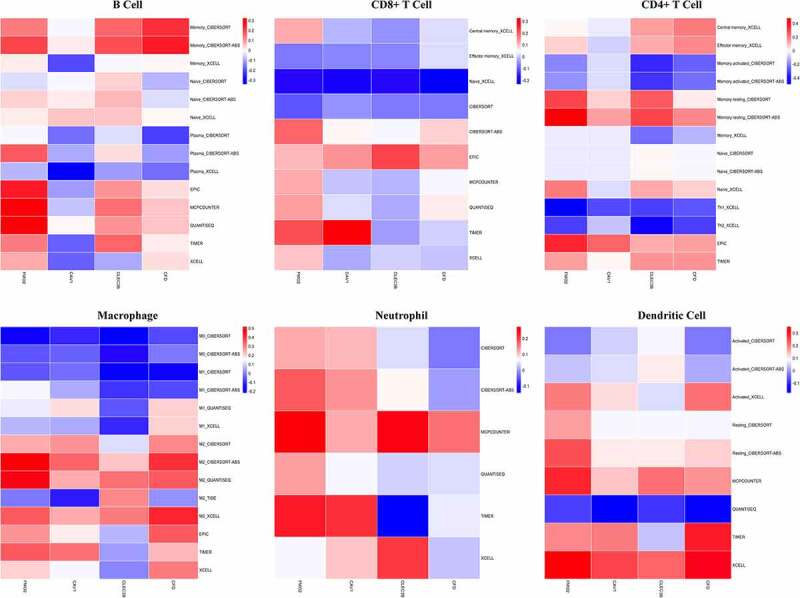
Correlations between biomarker expression and tumor infiltration with different calculation methods

Scatterplots showed the expression level of FMO2 was positively associated with all six types of immune infiltration. Moreover, CAV1, CFD, CLEC3B and FMO2 expression level was all positively related to macrophage. Overall, closely positive correlation was observed between biomarker expression and tumor infiltration, especially for CAV1, CFD and FMO2 (Figure 6). Therefore, we performed further correlation analysis with adjustment of tumor purity as the expression showed significant association with it. As to different methods of infiltration calculation, the expression of FMO2 was positively related to B cell and neutrophil. Naïve CD8 + T cell computed by the XCELL and macrophage calculated by the CIBERSORT were negatively associated with biomarker expression (Figure 7, Table S1).

## Discussion

The present work examined 5 GEO datasets and discovered 67 DEGs, among which, 15 showed up-regulation and 52 showed down-regulation. The top 3 biological process terms were extracellular structure organization, regulation of peptidase activity and fat cell differentiation, and in terms of KEGG pathways, the top three enriched pathways were phagosome, malaria and ECM-receptor interaction. The above functional annotation analysis shed more light on the molecular mechanism underlying LUAD occurrence. Firstly, macrophages can strongly destruct tumors by means of antibody-dependent phagocytosis. It is indicated that macrophage phagocytosis represents an important mechanism by which numerous antibodies can be applied in cancer treatment [18,19]. Malaria is also discovered to be related to tumors. According to epidemiological study, the malaria incidence shows negative correlation with mortality rates of LC, breast cancer (BC) and colorectal cancer(CRC) [20]. Studies have shown that some antimalarial drugs exhibit antitumor activities and are used to treat leukemia, osteoarthritis, renal cell carcinoma (RCC), CRC, BC, NSCLC, hepatoma and prostate cancer (PCa) [21,22]. The mechanism may involve the stimulation of the immune responses and inhibition of key pathways in tumor progress [23,24]. It has been confirmed that specific cell-cell and cell-extracellular matrix (ECM) interactions plastic plays a vital role in the invasion and metastasis of cancer cells [25], the degradation of ECM and basement membrane contributes to cell invasion metastasis [26,27]. At last, Our research revealed that PPARG was the most significant transcriptional regulatory approach by enrichment analysis based on the TRRUST database, a case-control study shows that PPARG c.1347 C > T polymorphism was associated with risk of NSCLC [28]. In short, all these results are consistent with our findings. The above results shed new lights on mechanism study and treatment strategy for LUAD.

CAV1, CFD, FMO2 and CLEC3B were eventually selected as independent prognostic biomarkers, and they were correlated with clinical outcomes of LUAD patients. The expression of each biomarker markedly decreased within tumor tissues in comparison with the matched non-carcinoma tissues (P < 0.001). More critically, Scatterplots showed the expression level of four biomarkers was positively associated with all different types of immune infiltration.

Caveolins(including CAV1) are essential for caveola formation, previous study has demonstrated that CAV1 is involved in mechanically regulating the extracellular environment as well as tumor metastasis and invasion. It has been confirmed that remodeling of the ECM by CAV1 is important for the architecture of normal organs, especially those that are rich in ECM fibers [29,30], and fibroblast expression of CAV1 favors directional migration and invasiveness of carcinoma cells in vitro [31]. However, as revealed by a cell proliferation assay, the over-expression of DCN and CAV1 markedly suppresses NSCLC cell proliferation [32], its role in invasion and migration of lung adenocarcinoma needs further study. Besides, LUAD cases who have increased CAV1 expression are found to have reduced lifespan, which is consistent with our findings [33].

Complement proteins are suggested to trigger the invasion of cancer by promoting EMT, degrading ECM and inducing growth factors as well as chemotactic stimuli [34,35]. In addition, complement activation is suggested as part of the anti-cancer immune surveillance in the body, and CFD plays an important role in activating the alternative pathway [36]. Besides, it is discovered that CFD is the same as adipokine adipsin that is expressed within macrophages/monocytes [37], and it may be involved in cancer-associated weight loss [38].

FMO2, a kind of NADPH-dependent enzyme, can catalyze substrate oxygenation [39], yet its function in tumorigenesis remains unknown. A bioinformatics analysis of adenocarcinoma found that FMO2 might have tumor suppressor effects in lung adenocarcinoma [40]. FMO2 was found as under-expressed genes in pre-invasive and invasive ductal breast carcinoma [41]. However, FMO2 was significantly upregulated in oral squamous cell carcinoma of early disease stages [42].

CLEC3B can encode a protein localized within cell plasma, exosomes as well as extracellular matrix (ECM), and it is related to tumor metastasis and invasion [43,44]. In LC cases, CLEC3B expression is markedly down-regulated relative to controls, which is related to LC prognostic outcome as well as TNM stage, suggesting that it is a LC risk factor. The present work suggested that, CLEC3B expression was positively correlated with immune cell infiltrating degree within SCC [45]. The above findings conform to our observations.

Recently, tumor immunotherapy has been rapidly developed, and it is found that the immune system plays an important part in LC genesis and development [46,47]. In addition, the immune infiltration within tumor microenvironment (TME) exerts an important part in tumor genesis and progression, which affects LC survival [7,48,49]. Various immunocyte infiltrates in NSCLC, including B cells, T cells, natural killer (NK) cells macrophages, as well as dendritic cells (DCs). In comparison with distal lung tissues, the levels of diverse immunocytes change in LC tissues [50]. It is previously suggested that, the elevated infiltrating levels of CD8 + T cells, M0 macrophages and acking memory B cells were related to the dismal prognostic outcome in LUAD [51,52]. On the other hand, tumor-associated macrophages (TAMs) exert a vital part in tumor development, anticancer immunity suppression together with dissemination [38]. Our study showed CAV1, CFD, CLEC3B and FMO2 expression level was all positively related to macrophage. A previous study demonstrates that macrophage CAV1 signaling is critical for metastasis, CAV1 in metastasis-associated macrophages (MAMs) specifically restrains vascular endothelial growth factor A/vascular endothelial growth factor receptor 1 (VEGF-A/VEGFR1) signaling and its downstream effectors [53]. Our study provides new insights into the immune mechanism of LUAD, but the role of other biomarkers in the immune invasion of lung adenocarcinoma cells needs further study.

## Conclusion

In conclusion, our study has identified a set of immune-related biomarkers for prognosis of lung adenocarcinoma. which could provide new ideas for immunotherapy of LUAD. However, some of the functions of these genes are still unclear, and more studies are needed to explore the molecular mechanisms of the new genes in LUAD.

## Supplementary Material

Supplemental Material

## Highlights

1. CAV1, CFD, FMO2 and CLEC3B can be used as independent prognostic biomarkers of LUAD.
2. A positive correlation was observed between biomarker expression and all different types of immune infiltration.
