# Supplementary material for: The screening of immune-related biomarkers for prognosis of lung adenocarcinoma
Source: Bioengineered. 2021 Apr 17;12(1):1273–85. doi: 10.1080/21655979.2021.1911211 (PMC8806236; doi:10.1080/21655979.2021.1911211)
Supplement: Supplemental Material [file KBIE_A_1911211_SM8104.zip › fig s1 caption.rtf]

Figure S1 LASSO Cox regression plot. (A) Partial likelihood deviance plot; (B) LASSO coefficient profile plot. The colorful curves stand for the LASSO coefficient profiles of diverse features as a function of the ln lambda sequence
