# Supplementary material for: The screening of immune-related biomarkers for prognosis of lung adenocarcinoma
Source: Bioengineered. 2021 Apr 17;12(1):1273–85. doi: 10.1080/21655979.2021.1911211 (PMC8806236; doi:10.1080/21655979.2021.1911211)
Supplement: Supplemental Material [file KBIE_A_1911211_SM8104.zip › Table S1.docx]

Table S1 Correlations between biomarker expression and tumor infiltration with different calculation methods

| Immune infiltrates | CAV1 | | CFD | | CLEC3B | | FMO2 | |
| --- | --- | --- | --- | --- | --- | --- | --- | --- |
|  | Partial Correlation | P.adjust | Partial Correlation | P.adjust | Partial Correlation | P.adjust | Partial Correlation | P.adjust |
| B cell memory_CIBERSORT | -0.014 | 0.843 | 0.222 | 0.000 | 0.161 | 0.002 | 0.129 | 0.013 |
| B cell memory_CIBERSORT-ABS | 0.015 | 0.839 | 0.257 | 0.000 | 0.190 | 0.000 | 0.195 | 0.000 |
| B cell memory_XCELL | -0.201 | 0.000 | 0.003 | 0.967 | -0.021 | 0.760 | 0.005 | 0.950 |
| B cell naive_CIBERSORT | -0.018 | 0.797 | -0.083 | 0.148 | 0.043 | 0.510 | -0.036 | 0.584 |
| B cell naive_CIBERSORT-ABS | 0.015 | 0.835 | -0.048 | 0.442 | 0.065 | 0.284 | 0.037 | 0.564 |
| B cell naive_XCELL | 0.052 | 0.411 | 0.004 | 0.960 | 0.050 | 0.427 | 0.018 | 0.808 |
| B cell plasma_CIBERSORT | -0.156 | 0.003 | -0.218 | 0.000 | -0.040 | 0.542 | -0.016 | 0.830 |
| B cell plasma_CIBERSORT-ABS | -0.093 | 0.098 | -0.103 | 0.062 | 0.031 | 0.643 | 0.176 | 0.000 |
| B cell plasma_XCELL | -0.341 | 0.000 | -0.158 | 0.002 | -0.103 | 0.064 | -0.083 | 0.140 |
| B cell_EPIC | -0.108 | 0.049 | 0.024 | 0.724 | 0.103 | 0.066 | 0.257 | 0.000 |
| B cell_MCPCOUNTER | -0.067 | 0.262 | 0.057 | 0.349 | 0.143 | 0.006 | 0.313 | 0.000 |
| B cell_QUANTISEQ | -0.002 | 0.985 | 0.050 | 0.422 | 0.109 | 0.048 | 0.324 | 0.000 |
| B cell_TIMER | -0.176 | 0.001 | 0.006 | 0.943 | 0.159 | 0.002 | 0.134 | 0.010 |
| B cell_XCELL | -0.180 | 0.000 | 0.019 | 0.785 | -0.094 | 0.099 | 0.076 | 0.181 |
| Macrophage M0_CIBERSORT | -0.102 | 0.067 | -0.065 | 0.278 | -0.218 | 0.000 | -0.183 | 0.000 |
| Macrophage M0_CIBERSORT-ABS | -0.030 | 0.660 | -0.004 | 0.961 | -0.143 | 0.006 | -0.048 | 0.435 |
| Macrophage M1_CIBERSORT | -0.014 | 0.847 | -0.181 | 0.000 | -0.189 | 0.000 | -0.045 | 0.476 |
| Macrophage M1_CIBERSORT-ABS | 0.059 | 0.330 | -0.069 | 0.245 | -0.089 | 0.122 | 0.142 | 0.006 |
| Macrophage M1_QUANTISEQ | 0.188 | 0.000 | 0.194 | 0.000 | -0.051 | 0.421 | 0.122 | 0.021 |
| Macrophage M1_XCELL | 0.057 | 0.357 | 0.207 | 0.000 | -0.111 | 0.044 | 0.069 | 0.235 |
| Macrophage M2_CIBERSORT | 0.278 | 0.000 | 0.286 | 0.000 | 0.113 | 0.040 | 0.240 | 0.000 |
| Macrophage M2_CIBERSORT-ABS | 0.333 | 0.000 | 0.404 | 0.000 | 0.222 | 0.000 | 0.516 | 0.000 |
| Macrophage M2_QUANTISEQ | 0.239 | 0.000 | 0.345 | 0.000 | 0.316 | 0.000 | 0.478 | 0.000 |
| Macrophage M2_TIDE | -0.128 | 0.016 | 0.031 | 0.637 | 0.289 | 0.000 | -0.013 | 0.866 |
| Macrophage M2_XCELL | 0.241 | 0.000 | 0.427 | 0.000 | 0.310 | 0.000 | 0.348 | 0.000 |
| Macrophage_EPIC | 0.176 | 0.001 | 0.354 | 0.000 | 0.072 | 0.231 | 0.271 | 0.000 |
| Macrophage_TIMER | 0.312 | 0.000 | 0.227 | 0.000 | 0.046 | 0.475 | 0.350 | 0.000 |
| Macrophage_XCELL | 0.140 | 0.008 | 0.298 | 0.000 | 0.003 | 0.971 | 0.200 | 0.000 |
| Dendritic cell activated_CIBERSORT | 0.046 | 0.476 | -0.021 | 0.757 | 0.082 | 0.161 | -0.027 | 0.700 |
| Dendritic cell activated_CIBERSORT-ABS | 0.061 | 0.321 | 0.014 | 0.845 | 0.104 | 0.063 | 0.038 | 0.550 |
| Dendritic cell activated_XCELL | 0.111 | 0.041 | 0.210 | 0.000 | 0.060 | 0.333 | 0.201 | 0.000 |
| Dendritic cell resting_CIBERSORT | 0.080 | 0.170 | 0.081 | 0.158 | 0.077 | 0.194 | 0.169 | 0.001 |
| Dendritic cell resting_CIBERSORT-ABS | 0.100 | 0.073 | 0.119 | 0.028 | 0.101 | 0.070 | 0.234 | 0.000 |
| Dendritic cell_MCPCOUNTER | 0.132 | 0.013 | 0.173 | 0.001 | 0.208 | 0.000 | 0.282 | 0.000 |
| Dendritic cell_QUANTISEQ | -0.178 | 0.000 | -0.158 | 0.002 | -0.093 | 0.103 | -0.078 | 0.165 |
| Dendritic cell_TIMER | 0.195 | 0.000 | 0.291 | 0.000 | 0.039 | 0.552 | 0.189 | 0.000 |
| Dendritic cell_XCELL | 0.247 | 0.000 | 0.308 | 0.000 | 0.221 | 0.000 | 0.350 | 0.000 |
| Neutrophil_CIBERSORT | 0.114 | 0.036 | -0.025 | 0.707 | 0.046 | 0.474 | 0.119 | 0.025 |
| Neutrophil_CIBERSORT-ABS | 0.140 | 0.008 | 0.002 | 0.980 | 0.067 | 0.265 | 0.179 | 0.000 |
| Neutrophil_MCPCOUNTER | 0.119 | 0.026 | 0.164 | 0.001 | 0.252 | 0.000 | 0.273 | 0.000 |
| Neutrophil_QUANTISEQ | 0.065 | 0.286 | 0.043 | 0.497 | 0.037 | 0.576 | 0.130 | 0.012 |
| Neutrophil_TIMER | 0.208 | 0.000 | 0.055 | 0.371 | -0.142 | 0.007 | 0.227 | 0.000 |
| Neutrophil_XCELL | 0.104 | 0.060 | 0.032 | 0.632 | 0.204 | 0.000 | 0.065 | 0.266 |
| T cell CD4+ central memory_XCELL | -0.041 | 0.526 | 0.202 | 0.000 | 0.138 | 0.009 | -0.007 | 0.931 |
| T cell CD4+ effector memory_XCELL | -0.071 | 0.235 | 0.182 | 0.000 | 0.127 | 0.018 | 0.067 | 0.246 |
| T cell CD4+ memory activated_CIBERSORT | -0.060 | 0.327 | -0.245 | 0.000 | -0.340 | 0.000 | -0.193 | 0.000 |
| T cell CD4+ memory activated_CIBERSORT-ABS | -0.053 | 0.397 | -0.240 | 0.000 | -0.335 | 0.000 | -0.181 | 0.000 |
| T cell CD4+ memory resting_CIBERSORT | 0.052 | 0.412 | 0.030 | 0.650 | 0.248 | 0.000 | 0.293 | 0.000 |
| T cell CD4+ memory resting_CIBERSORT-ABS | 0.131 | 0.014 | 0.182 | 0.000 | 0.286 | 0.000 | 0.478 | 0.000 |
| T cell CD4+ memory_XCELL | -0.033 | 0.623 | -0.122 | 0.024 | -0.227 | 0.000 | -0.046 | 0.462 |
| T cell CD4+ naive_CIBERSORT | -0.043 | 0.502 | -0.022 | 0.745 | -0.005 | 0.948 | -0.039 | 0.539 |
| T cell CD4+ naive_CIBERSORT-ABS | -0.043 | 0.502 | -0.022 | 0.745 | -0.005 | 0.948 | -0.039 | 0.539 |
| T cell CD4+ naive_XCELL | -0.050 | 0.428 | 0.069 | 0.252 | 0.114 | 0.038 | 0.206 | 0.000 |
| T cell CD4+ Th1_XCELL | -0.299 | 0.000 | -0.276 | 0.000 | -0.317 | 0.000 | -0.494 | 0.000 |
| T cell CD4+ Th2_XCELL | -0.100 | 0.071 | -0.308 | 0.000 | -0.455 | 0.000 | -0.316 | 0.000 |
| T cell CD4+_EPIC | 0.231 | 0.000 | 0.139 | 0.008 | 0.118 | 0.029 | 0.352 | 0.000 |
| T cell CD4+_TIMER | -0.003 | 0.972 | 0.165 | 0.001 | 0.148 | 0.005 | 0.143 | 0.005 |
| T cell CD8+ central memory_XCELL | -0.011 | 0.880 | 0.013 | 0.852 | -0.048 | 0.448 | 0.121 | 0.022 |
| T cell CD8+ effector memory_XCELL | -0.058 | 0.349 | 0.023 | 0.736 | -0.056 | 0.374 | -0.075 | 0.191 |
| T cell CD8+ naive_XCELL | -0.198 | 0.000 | -0.233 | 0.000 | -0.170 | 0.001 | -0.166 | 0.001 |
| T cell CD8+_CIBERSORT | -0.047 | 0.454 | -0.073 | 0.219 | -0.069 | 0.256 | -0.099 | 0.068 |
| T cell CD8+_CIBERSORT-ABS | 0.047 | 0.456 | 0.091 | 0.108 | 0.046 | 0.473 | 0.185 | 0.000 |
| T cell CD8+_EPIC | 0.147 | 0.005 | 0.126 | 0.019 | 0.225 | 0.000 | 0.104 | 0.055 |
| T cell CD8+_MCPCOUNTER | 0.000 | 1.000 | 0.043 | 0.498 | -0.012 | 0.869 | 0.120 | 0.023 |
| T cell CD8+_QUANTISEQ | 0.022 | 0.754 | 0.067 | 0.266 | -0.022 | 0.752 | 0.127 | 0.015 |
| T cell CD8+_TIMER | 0.326 | 0.000 | 0.003 | 0.973 | -0.034 | 0.606 | 0.212 | 0.000 |
| T cell CD8+_XCELL | -0.028 | 0.680 | 0.000 | 1.000 | 0.007 | 0.926 | 0.093 | 0.091 |
