# Supplementary figures and images for: The screening of immune-related biomarkers for prognosis of lung adenocarcinoma
Source: Bioengineered. 2021 Apr 17;12(1):1273–85. doi: 10.1080/21655979.2021.1911211 (PMC8806236; doi:10.1080/21655979.2021.1911211)

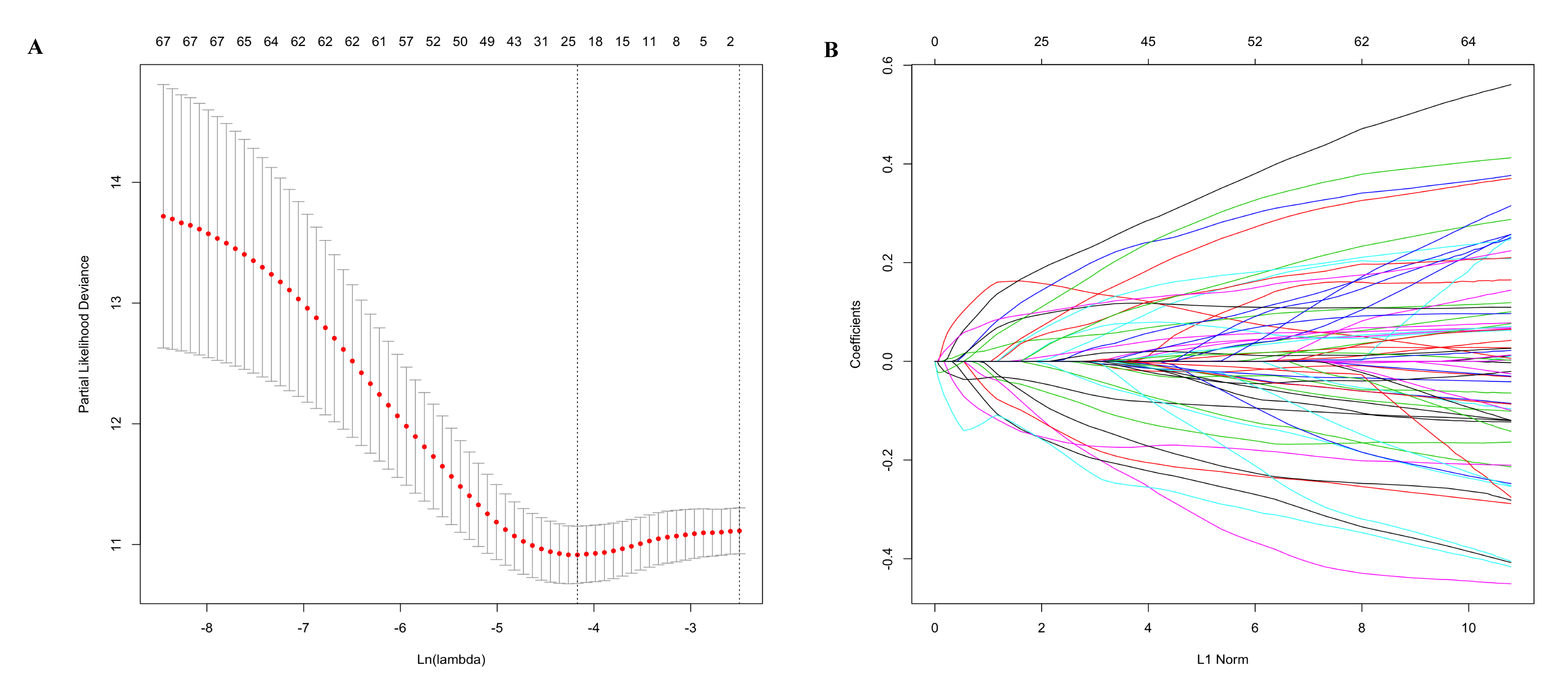

Supplement: Supplemental Material [file KBIE_A_1911211_SM8104.zip › Figure S1.tif]
